# Cell-Penetrating Peptide and siRNA-Mediated Therapeutic Effects on Endometriosis and Cancer In Vitro Models

**DOI:** 10.3390/pharmaceutics13101618

**Published:** 2021-10-05

**Authors:** Kristina Kiisholts, Kaido Kurrikoff, Piret Arukuusk, Ly Porosk, Maire Peters, Andres Salumets, Ülo Langel

**Affiliations:** 1 Institute of Technology, University of Tartu, 50411 Tartu, Estonia; kristina.kiisholts@gmail.com (K.K.); kaido.kurrikoff@ut.ee (K.K.); piret.arukuusk@gmail.com (P.A.); lyparnaste@gmail.com (L.P.); 2 Competence Centre on Health Technologies, 50411 Tartu, Estonia; maire.peters@ut.ee (M.P.); andres.salumets@ccht.ee (A.S.); 3 Department of Obstetrics and Gynecology, Institute of Clinical Medicine, University of Tartu, 50406 Tartu, Estonia; 4 Institute of Genomics, University of Tartu, 51010 Tartu, Estonia; 5 Division of Obstetrics and Gynecology, Department of Clinical Science, Intervention and Technology (CLINTEC), Karolinska Institute, 14152 Stockholm, Sweden; 6 Department of Biochemistry and Biophysics, Stockholm University, 10691 Stockholm, Sweden

**Keywords:** cell-penetrating peptides, siRNA, nanoparticles, endometriosis, cancer

## Abstract

Gene therapy is a powerful tool for the development of new treatment strategies for various conditions, by aiming to transport biologically active nucleic acids into diseased cells. To achieve that goal, we used highly potential delivery vectors, cell-penetrating peptides (CPPs), as oligonucleotide carriers for the development of a therapeutic approach for endometriosis and cancer. Despite marked differences, both of these conditions still exhibit similarities, like excessive, uncoordinated, and autonomous cellular proliferation and invasion, accompanied by overlapping gene expression patterns. Thus, in the current study, we investigated the therapeutic effects of CPP and siRNA nanoparticles using in vitro models of benign endometriosis and malignant glioblastoma. We demonstrated that CPPs PepFect6 and NickFect70 are highly effective in transfecting cell lines, primary cell cultures, and three-dimensional spheroids. CPP nanoparticles are capable of inducing siRNA-specific knockdown of therapeutic genes, ribonucleotide reductase subunit M2 (RRM2), and vascular endothelial growth factor (VEGF), which results in the reduction of in vitro cellular proliferation, invasion, and migration. In addition, we proved that it is possible to achieve synergistic suppression of endometriosis cellular proliferation and invasion by combining gene therapy and hormonal treatment approaches by co-administering CPP/siRNA nanoparticles together with the endometriosis-drug danazol. We suggest a novel target, RRM2, for endometriosis therapy and as a proof-of-concept, we propose a CPP-mediated gene therapy approach for endometriosis and cancer.

## 1. Introduction

Cancer represents a second leading source of mortality worldwide after cardiac pathologies and is considered to exhibit the highest clinical, social, and economic burden among all human diseases [1]. Despite several radical interventions, like surgical resection of solid tumors, radiotherapy, and administration of chemotherapeutics, the prognosis remains poor for many cancer patients. What is more, current therapies often induce severe toxicity in normal tissues, thus alternative therapeutics are needed and actively searched for [2]. Endometriosis, on the other hand, is a hormone-dependent neuroinflammatory gynecological disease that is generally not life-threatening. Regardless, it has a significant influence on the life quality of up to 10% of women of reproductive age [3]. The disorder is characterized by the presence of endometrial tissue outside the uterine cavity and it causes chronic pelvic pain, dysmenorrhea or dyspareunia, fatigue, and infertility. The current treatment strategies include the laparoscopic excision of the lesions and/or pharmacological therapy, primarily modulating the patients’ hormonal balance. The drawback of the surgical approach is its invasiveness and the high recurrence rate of lesions. Hormonal therapy, although being effective in controlling the chronic pain, does not eliminate the lesions and is not suitable for women trying to conceive [4]. One of the widely used drugs in endometriosis treatment is a synthetic androgenic hormone, danazol, that has proved to be very effective in pain alleviation and inducing lesion regression [5]. Nevertheless, its use is limited as it causes severe hyperandrogenic side effects in the case of systemic administration, including abnormal changes in hormonal balance, disturbance of menstrual cycle, and overall masculine effects [5]. Therefore, there is an urgent need for the development of new tools for the treatment of endometriosis.

Gene therapy could serve as a suitable treatment strategy for both conditions, since apart from systemic pharmacological treatment, it specifically targets genes that are abnormally expressed in the diseased tissue. For example, the use of the RNA interference pathway for highly specific silencing of overexpressed genes by delivering miRNA/siRNA into cells has been considered as one of the most promising gene therapy approaches for decades already [6]. Gene expression patterns in endometriotic lesions have been widely studied, however, the disease is highly heterogenic and, therefore, only a few potential target gene candidates have been suggested [7]. One of them is vascular endothelial growth factor (VEGF), which is overexpressed in endometriotic lesions compared to healthy tissues and has been successfully used as a target in preclinical endometriosis therapy [8]. VEGF is considered to be a key factor in the process of neovascularization; therefore, it is also a widely known target gene in solid tumor therapy [9]. Furthermore, another gene, ribonucleotide reductase, has been shown to be crucial during the proliferation of cancer cells, and the same applies for VEGF, a variety of their different inhibitors are being used in clinics or undergoing clinical trials for tumor growth inhibition [9,10]. Although endometriosis is not considered malignant, it has been shown to exhibit similar behavior to cancer, e.g., increased invasion and migration of cells or inflammation and angiogenesis in tissues [11], accompanied by cancer driver gene expression in the endometriotic lesions [12]. Therefore, the potential role of silencing ribonucleotide reductase subunit M2 (RRM2) in endometriosis therapy has been investigated in this work, in addition to VEGF-A as a therapeutic siRNA target.

For successful gene therapy, nucleic acids have to reach their intracellular targets, and for that, highly efficient delivery vectors that are harmless to healthy tissue and do not cause severe immune reactions are needed. Cell-penetrating peptides (CPPs) are highly potential non-viral vectors that are generally considered safe, easy to synthesize and modify, and enable simple non-covalent CPP/siRNA nanoparticle formulation [13]. One highly effective CPP for oligonucleotide delivery is PepFect6 (PF6), which has also been shown to be successful in transfecting primary cells [14]. Furthermore, PF6 has proved to be non-toxic in vitro and has not shown immunogenicity in vivo [15]. Another CPP, the recently designed NickFect70 (NF70) [16], has been shown to be as effective as PF6 in siRNA delivery. 

In this study, we show the high potential of using cell-penetrating peptides PF6 and NF70 as siRNA delivery vectors for the treatment of cancer and endometriosis. Both CPPs were capable of successfully transfecting two- (2D) and three-dimensional (3D) cell cultures. Moreover, successful delivery of therapeutic siRNAs against RRM2 (siRRM2) and VEGF (siVEGF) led to downregulation of both genes, accompanied by the phenotypic changes of tumor and endometriotic cells. We have demonstrated disease-specific therapeutic effects by reducing cell migration from the 3D tumor model and synergistic reduction of endometriotic cell number and invasiveness after therapeutic CPP/siRNA nanoparticle (NP) and danazol co-treatment.

## 2. Materials and Methods

### 2.1. Solid Phase Peptide Synthesis

Peptides were synthesized on an automated peptide synthesizer (Biotage Initiator+ Alstra, Uppsala, Sweden) using the fluorenylmethyloxycarbonyl (Fmoc) solid-phase peptide synthesis strategy with Rink-amide ChemMatrix resin to obtain C-terminally amidated peptides. As coupling reagents, HOBT/HBTU in DMF and DIEA as an activator base were used. The fatty acid (5 eq.) was coupled manually to the N-terminus of the peptide at room temperature overnight. 

The synthesis of NF70 is described in Porosk et al. [16]. Briefly, for continuing the synthesis from the side chain amino group of Orn9, Boc-l-Orn(Fmoc)-OH (Iris Biotech, Marktredwitz, Germany) was used and synthesis was continued through the side chain amino group. Arachidic acid (Acros Organics, Carlsbad, CA, USA) was coupled to the N-terminus of the peptide as described above. 

PF6 was synthesized as described in El Andaloussi et al. [14]. Briefly, Fmoc-Lys (Fmoc) (Iris Biotech, Marktredwitz, Germany) was used for lysine tree and chloroquine analogues (trifluoromethyl quinoline derivative) were coupled to it over succinic anhydride (Sigma-Aldrich, Taufkirchen, Germany). Stearic acid (Sigma-Aldrich, Taufkirchen, Germany) was coupled to the N-terminus of the peptide. 

Cleavage was performed with TFA, 2.5% triisopropylsilane, and 2.5% water for 3 h at room temperature. Peptides were purified by HPLC on a C4 column (Phenomenex Jupiter C4, 5 μm, 300 Å, 250 × 10 mm) using acetonitrile/water gradient containing 0.1% TFA. The molecular weight of the peptides was analyzed by MALDI-TOF (Brucker Microflex LT/SH, Billerica, MA, USA). The HPLC and MALDI-TOF graphs for PF6 and NF70 are shown in Figure S1. The concentration of the peptides was determined based on dilutions of accurately weighed substances and absorption of tyrosine, where applicable.

### 2.2. Endometriotic Sample Collection and Stromal Cell Isolation

Primary peritoneal and ovarian endometriotic stromal cells were isolated from patients’ endometriotic lesions, which were collected from women undergoing laparoscopy at the Tartu University Hospital Women’s Clinic. Patient sample collection was approved by the Research Ethics Committee of the University of Tartu (approval No. 276/M-13, Tartu, Estonia) and informed written consent was obtained from the participants. Tissues (one peritoneal lesion and two ovarian endometriomas) from three reproductive age (25 ± 1.5 years of age) patients suffering from stage III–IV endometriosis, were used. The patients had not received any hormonal medication three months before the recruitment. Primary stromal cells were isolated from endometriosis biopsies as described in Kasvandik et al. [17]. Briefly, endometriotic tissues were washed with Dulbecco’s Modified Eagle’s Medium (DMEM, Gibco, Thermo Fisher Scientific, Waltham, MA, USA) and dissociated using 0.5% collagenase (Sigma-Aldrich, St. Louis, MO, USA). The resulting tissue suspension was filtered through a 50 μm nylon mesh (Corning, Corning, NY, USA) and cells were resuspended in cell culture medium. The epithelial glands were eliminated by repeated sedimentation followed by selective adherence of stromal cells to culture dishes and discarding remaining non-adherent epithelial cells.

### 2.3. Cell Culture

Endometriotic primary cells were cultured in 1:1 mixture of DMEM (Gibco, Thermo Fisher Scientific, Waltham, MA, USA) and Ham’s F-12 (Corning, Corning, NY, USA), while U-87MG human glioblastoma (#HTB-14, ATCC, Manassas, VA, USA) and HT1080 human fibrosarcoma (#CCL-121, ATCC, Manassas, VA, USA) cells were cultured in DMEM (Gibco, Thermo Fisher Scientific, Waltham, MA, USA). U-87MG cells were grown on 0.1% gelatin (Naxo, Tartu, Estonia)-coated plates. Unless specified separately, all media were supplemented with 10% fetal bovine serum (FBS, Sigma-Aldrich, St. Louis, MO, USA), 0.1 mmol/L MEM non-essential amino acids (Sigma-Aldrich, St. Louis, MO, USA), 1.0 mmol/L sodium pyruvate (Sigma-Aldrich, St. Louis, MO, USA), and 100 U/mL penicillin + 100 μg/mL streptomycin (Gibco, Thermo Fisher Scientific, Waltham, MA, USA). In addition, 0.25% trypsin-EDTA (Sigma-Aldrich, St. Louis, MO, USA) was used for cell detachment. Cells were grown at 37 °C, 5% CO_2_, in a humidified environment. 

For the formation of 3D spheroids, cells were seeded on Nunclon Sphera low-attachment 96U-well plates (Thermo Fisher Scientific, Waltham, MA, USA) and cultured by following previously described guidelines [18]. Briefly, the plates were left undisturbed for 48 h after seeding in order to facilitate uniform spheroid formation. To maintain the integrity of the spheroids, only 50% of culture media was replaced every other day. Since it has been shown that 3D spheroids can develop a necrotic core over time, it is most optimal to start the treatment for investigating therapeutic effects on the fourth day after seeding and using spheroids that have a diameter of approximately 400 µm [18]. The cell density during spheroid seeding was optimized to meet these requirements and was determined to be 2000 or 10,000 cells per well for tumor or endometriotic cells, respectively. 

### 2.4. siRNA Design, Nanoparticle Formation, and Transfection

The siRNA (Microsynth, Balgach, Switzerland) sequences are listed in Table S1. siRRM2 was designed as a Dicer substrate duplex RNA using the Integrated DNA Technologies DsiRNA design tool and the dose used for all of the siRNA transfections was chosen based on the dose titration results of this custom siRRM2 (Figure S2). Sequences of siVEGF against VEGF-A mRNA [19], siLuc [14], and siLuc2_Cy5 [20] have previously been published. Throughout the work, siLuc was used as a negative control irrelevant siRNA, referred to as siMock, whereas fluorescently labelled siLuc2_Cy5 was referred to as siMock_Cy5. 

Monolayer cells seeded on 24-well plates (Greiner Bio-One, Kremsmünster, Austria) were transfected when they reached approximately 50% confluency, while 3D spheroids were transfected on the fourth day after seeding. CPP/siRNA nanoparticles were formed in a non-covalent manner at molar ratio 40 (CPP/siRNA) by mixing the peptide with siRNA in MilliQ water (pH 5.3–6.3) followed by incubation at room temperature for 45 min before transfection. siRNA final concentration in cell culture media was 100 nM for 2D and 200 nM for 3D cultures. As a control group, pure siRRM2 or siVEGF without a delivery vector was added to cells at equivalent concentrations to nanoparticle transfection. As a positive control for siRNA delivery, commercially available Lipofectamine RNAiMAX Transfection Reagent (Invitrogen, Thermo Fisher Scientific, Waltham, MA, USA) was used according to manufacturer’s protocol, adjusting the dose to match siRNA concentrations during transfection with CPPs. 

### 2.5. Gene Expression Analysis

Total mRNA was isolated from the cell cultures 48 h after transfection using TRIzol Reagent (Invitrogen, Thermo Fisher Scientific, Waltham, MA, USA) and cDNA was synthesized using Superscript IV Reverse Transcriptase (Invitrogen, Thermo Fisher Scientific, Waltham, MA, USA) according to the manufacturer’s protocol. For qRT-PCR, EvaGreen qPCR Supermix reagent (Solis BioDyne, Tartu, Estonia) was used according to the manufacturer’s protocol and the experiment was conducted using a ViiA 7 Real-Time PCR System (Applied Biosystems, Thermo Fisher Scientific, Waltham, MA, USA). All primer sequences are listed in Table S2. The relative gene expression levels were normalized according to the level of *GAPDH* housekeeper gene and the obtained data were analyzed using 2^−ΔΔCT^ method by comparing all treatment groups with relevant pure siRNA-treated groups.

### 2.6. Immunocytochemistry

U-87MG tumor and endometriotic primary cells were seeded on Nunc Lab-Tek 8-well coverslips (Thermo Fisher Scientific, Waltham, MA, USA) and when they had become about 50% confluent, cells were transfected with CPP/siRNA NPs. The cells were fixed with 4% formaldehyde (Naxo, Tartu, Estonia) 48 h post-transfection. Nonspecific staining was reduced using species-specific blocking solution containing 0.25% TritonX-100 in PBS supplemented with 5% normal donkey serum (Sigma-Aldrich, St. Louis, MO, USA). Cells were incubated overnight with primary R2 antibody (#sc-10846, Santa Cruz, Dallas, TX, USA) against RRM2 in blocking solution (1:500), followed by 3 h incubation with Alexa488-labelled secondary antibody (#A-11055, Invitrogen, Thermo Fisher Scientific, Waltham, MA, USA) in blocking solution (1:1000). Nuclei were counterstained with DAPI (Sigma-Aldrich, St. Louis, MO, USA). Cells were visualized using an LSM710 confocal microscope and images were analyzed using ZEN microscope software (Zeiss, Oberkochen, Germany). 

### 2.7. Cell Cycle Analysis

Cells were detached from culture plates using 0.25% trypsin-EDTA (Sigma-Aldrich, St. Louis, MO, USA) 48 h after CPP/siRRM2 transfection. Next, 80% cold ethanol was used for fixing the cells, followed by a 30 min incubation in staining solution containing 2.5 μg/mL propidium iodide (Sigma-Aldrich, St. Louis, MO, USA) and 4 μg/mL RNase A (Thermo Fisher Scientific, Waltham, MA, USA). The cell cycle was analyzed using BD LSR-II (BD Biosciences, Franklin Lakes, NJ, USA) flow cytometer.

### 2.8. Invasion Assessment

Invasion assay was carried out on Matrigel invasion chambers (Corning, Corning, NY, USA) according to the manufacturer’s protocol. Briefly, 48 h after transfection with CPP/siRNA NPs, cells were detached from 24-well plates using 0.25% trypsin-EDTA (Sigma-Aldrich, St. Louis, MO, USA) and 25,000 cells were transferred to the Matrigel-coated inserts. Then, 24 h after the transfer, the regular media in the upper chamber was replaced with serum-free media. A 10% FBS-containing media was used as a chemoattractant in the lower wells. Next, 24 h after media change, non-invading cells were removed and cells on the lower surface of the membrane were fixed with ice-cold absolute methanol (Thermo Fisher Scientific, Waltham, MA, USA) and stained with 1% methylene blue (BioGnost, Zagreb, Croatia) and 1% eosin (BioGnost, Zagreb, Croatia). Cells were visualized and counted under a Nikon Eclipse TS100 inverted microscope.

### 2.9. 3D Spheroid Tissue Penetration

Spheroids were transfected with CPP NPs with Cy5 fluorescently labelled siRNA, and then 1.5 h later washed with regular culture media and transferred to Nunc Lab-Tek 8-well coverslips (Thermo Fisher Scientific, Waltham, MA, USA) using 1 mL ultra-low-retention pipette tips (Brand, Wertheim, Germany). Spheroids were kept at 37 °C in a 5% CO_2_ environment during the whole experiment and live-imaged under LSM710 confocal microscope (Zeiss, Oberkochen, Germany) at different time points (2, 4, and 6 h). Z-stack acquisition was used and mid-height section images are presented. Images were analyzed using ZEN microscope software (Zeiss, Oberkochen, Germany).

### 2.10. 3D Spheroid Migration Assay

Migration assay was adapted from previously described protocols [21,22]. In more detail, spheroids were transfected with different CPP/siRNA NPs 4 days after seeding. Then, 48 h after transfection, spheroids were transferred to the middle of Nunc Lab-Tek 8-well coverslips (Thermo Fisher Scientific, Waltham, MA, USA) previously coated with 0.1% gelatin (Naxo, Tartu, Estonia), one spheroid per well. A further 3 days after that, 3D cultures were fixed with 4% formaldehyde (Naxo, Tartu, Estonia) and stained with 1% methylene blue (BioGnost, Zagreb, Croatia) and 1% eosin (BioGnost, Zagreb, Croatia). Spheroids were visualized and photographed under stereomicroscope (Leica M165FC, Wetzlar, Germany). Images were analyzed and cell migration surface area was measured using ImageJ software.

Migration evaluation was the only assay, where HT1080 cells were used instead of U-87MG due to technical reasons. Namely, U-87MG cell migration from the spheroids was scattered and irregular, which did not allow the quantification of the results. Therefore, the U-87MG cell line was replaced with another 3D model available that had previously shown to exhibit regular cell migration patterns according to our experience.

### 2.11. CPP/Therapeutic siRNA and Danazol Co-Treatment

CPP/siRNA nanoparticles were applied on cells growing on the 24-well plate as usual, directly followed by the addition of danazol (Cayman Chemical, Ann Arbor, MI, USA) dissolved in 96% ethanol (Thermo Fisher Scientific, Waltham, MA, USA) at different final concentrations (10, 15, and 20 µM). The concentration of danazol and treatment duration was chosen relying on previously described results in the literature [23]. Then, 48 h post-treatment, cells were fixed with ice-cold absolute methanol (Thermo Fisher Scientific, Waltham, MA, USA) and stained with 1% methylene blue (BioGnost, Zagreb, Croatia). After washing the stained cells with PBS, methylene blue was eluted with 1 M HCl (Sigma-Aldrich, St. Louis, MO, USA) and its absorbance was measured at 650 nm for each individual sample. In case of invasion assessment, the cells were co-treated on 24-well plates and the invasive capability was measured as described previously in the methods’ invasion assessment section.

### 2.12. Statistical Analysis

Data were analyzed using Statistica software (StatSoft Europe, Hamburg, Germany) and results are expressed as mean ± SEM. One-way ANOVA was used to study differences among all treatments, except for the NP–danazol co-treatment studies, where two-way ANOVA was used. Tukey HSD test was performed for post hoc analysis and significance level was set at *p* < 0.05. GraphPad Prism was used for preparing the figures.

## 3. Results

### 3.1. CPP/siRNA NPs Mediate RRM2 and VEGF Knockdown and Cell Cycle Arrest in Tumor Cells

The physicochemical properties of the CPP/siRNA NPs, including size, shape, polydispersity index, etc., have been studied in our previous publications. The CPPs form stable, similarly spherical nanoparticles within the size range from 30–40 nm (TEM) or 100 to 200 nm (DLS) when associated with siRNA. The complex stability and packing properties have also been characterized accompanied by cytotoxicity testing in different immortalized and primary cells, generally exhibiting low toxicity at similar experimental conditions [14,16,24].

For cancer modelling, we used cells originating from one of the most common types of malignant central nervous system tumors, glioblastoma, which is also one of the most aggressive solid tumors with a five-year survival rate being only around 6.8% [25]. More specifically, we chose a widely used cell line in preclinical cancer therapy research, U-87MG.

**Figure 1:**
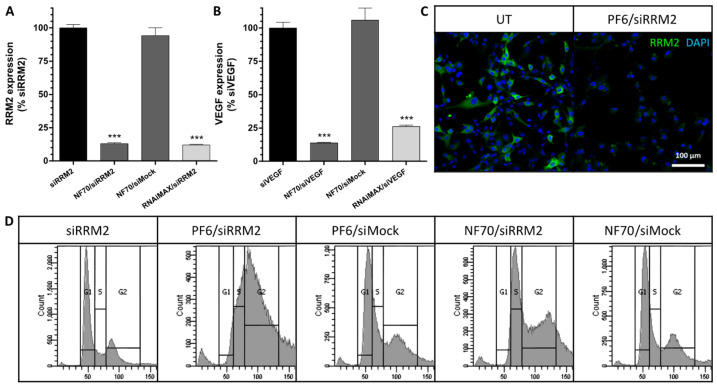
CPP/siRNA NPs induced therapeutic gene knockdown, RRM2 protein decrease, and cell cycle arrest in U-87MG cells. (**A**) *RRM2* and (**B**) *VEGF* gene expression levels in tumor cells measured with qRT-PCR. Data were analyzed by 2^−ΔΔCT^ method using *GAPDH* as an internal control and normalized to relevant pure siRNA groups. Error bars represent SEM, *** *p* < 0.001, one-way ANOVA, Tukey post hoc. (**C**) Representative immunocytochemical staining of RRM2 (green) in tumor cells counterstained with DAPI (blue); scale bar: 100 µm. (**D**) Representative flow cytometry graphs of propidium-iodide-stained tumor cells. All of the data (**A**–**D**) were collected 48 h after transfection from 3 independent experiments. UT, untreated.

RRM2 and VEGF are both well-known targets for anti-tumor therapy [9,10]. Hence, in order to validate the siRNAs and optimize the transfection conditions, gene expression evaluation was carried out in U-87MG tumor cells. All results were normalized to relevant pure siRNA treatments at equivalent concentrations to CPP-NP-treated groups. As expected, CPP nanoparticles with siRRM2 and siVEGF proved to mediate significant target gene knockdown (Figure 1A,B, respectively), whereas the downregulation of *VEGF* was slightly lower when using Lipofectamine RNAiMAX as a positive control for transfection reagent (Figure 1B). CPP/siMock NPs were used in parallel as a negative control and proved not to influence the gene expression levels (Figure 1A,B).

In order to test whether *RRM2* gene expression downregulation had also led to a decrease in the RRM2 protein amount and to validate and optimize the antibody against RRM2, immunocytochemical analysis of transfected U-87MG cells was performed. Indeed, knockdown of *RRM2* was accompanied by RRM2 protein level decrease in tumor cells after PF6/siRRM2 treatment compared to untreated (UT) cells (Figure 1C).

Ribonucleotide reductase is a crucial enzyme for DNA synthesis during cell proliferation, and while its M1 subunit is expressed constantly throughout the cell cycle, the M2 subunit is expressed solely during late G1 or early S-phase [26]. Hence, when RRM2 is efficiently knocked down, the cell cycle should be arrested in G1/S phase. We performed the flow cytometry analysis of propidium-iodide-stained U-87MG cells, which confirmed cell cycle arrest after CPP/siRRM2 treatment. NPs with mock siRNA, on the other hand, did not influence the normal cell cycle when compared to pure siRRM2-treated cells (Figure 1D).

### 3.2. CPP/siRNA NPs Mediate Target Gene Knockdown and Cell Cycle Arrest in Endometriotic Primary Cells

Endometriosis is a heterogenous disease with three different subtypes described—superficial peritoneal, ovarian, and deep infiltrating endometriosis. The most frequent anatomic site of endometriosis is considered to be the ovary, which is often accompanied by superficial lesions [27]. Since tumors and endometriotic lesions have been shown to exhibit similar traits [11,12], as a next step, we aimed to investigate the effect of applying the siRRM2 and siVEGF NP formulations on endometriotic cells. The heterogenicity of endometriosis is also described by the presence of two main cell types in the lesions, stromal cells and epithelial glands. Nevertheless, recent studies have shown that in a notable proportion of endometriotic lesions only stromal compartment is present [28]. Therefore, as a proof of concept, primary stromal cells derived from two different types of endometriosis, peritoneal and ovarian lesions, were used.

**Figure 2:**
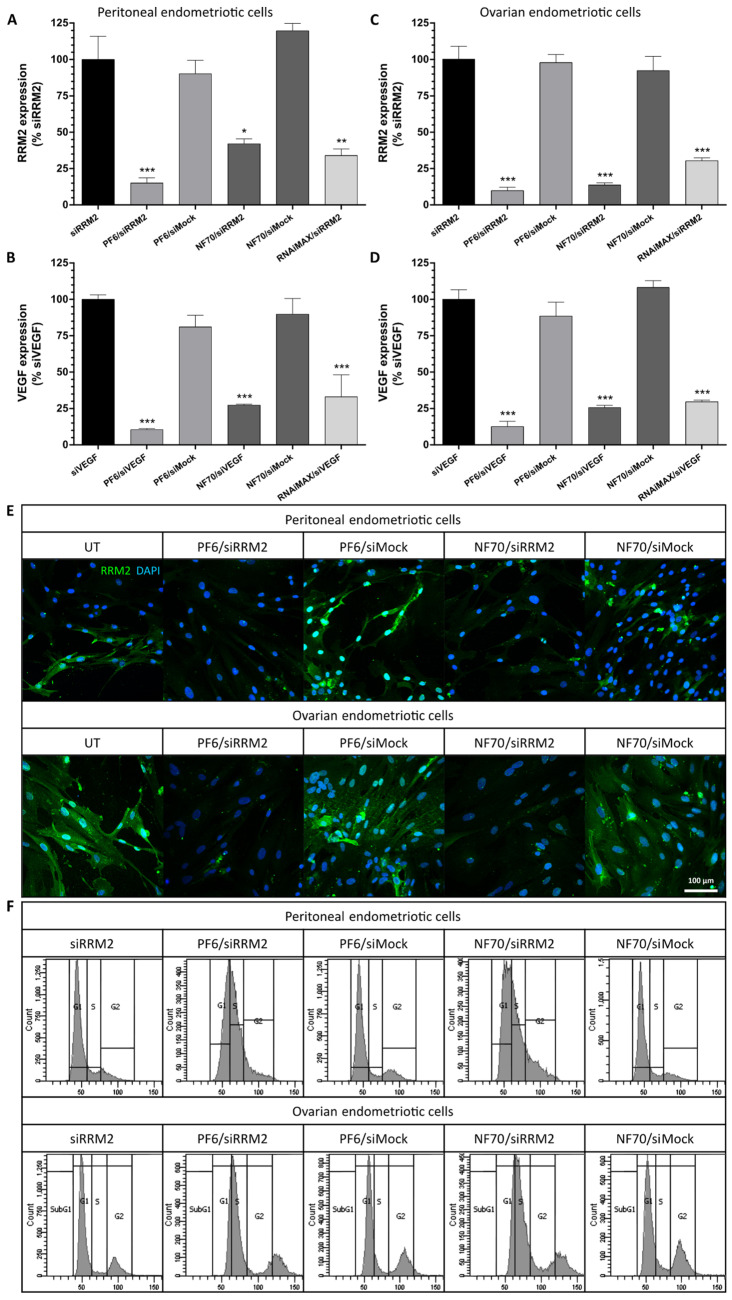
CPP/siRNA NPs induced therapeutic gene knockdown, RRM2 protein decrease, and cell cycle arrest in endometriotic cells. (**A**,**B**) *RRM2* and (**C**,**D**) *VEGF* gene expression levels measured in (**A**,**C**) peritoneal and (**B**,**D**) ovarian endometriotic cells using qRT-PCR. Data were analyzed by 2^−ΔΔCT^ method using *GAPDH* as an internal control and normalized to relevant pure siRNA groups. Error bars represent SEM of *n* = 7–8 individual samples from independent experiments. * *p* < 0.05, ** *p* < 0.01, *** *p* < 0.001, one-way ANOVA, Tukey post hoc. (**E**) Representative immunocytochemical staining of RRM2 (green) in endometriotic primary cells counterstained with DAPI (blue); scale bar: 100 µm. (**F**) Representative flow cytometry graphs of propidium-iodide-stained endometriotic peritoneal and ovarian cells. All of the data (**A**–**F**) were collected 48 h after transfection and representative images (**E**,**F**) are from 3 independent experiments. UT, untreated.

Endometriotic cells’ *RRM2* and *VEGF* gene expression analysis after CPP/siRNA treatment is shown in Figure 2A–D. Results revealed that PF6 and NF70 NPs are both capable of inducing significant target gene knockdown when compared to pure siRNA-treated cells in the case of both endometriosis subtypes. Lipofection efficacy remained mainly at a similar level to CPP NPs with relevant siRNAs, whereas CPP/siMock NPs did not influence gene expression levels.

Since we suggested RRM2 as a novel target for endometriosis therapy, we aimed to further investigate the effect of its downregulation to the cells. For that, we determined RRM2 protein levels after transfection by conducting immunocytochemical analysis of endometriotic cells. As a result, in addition to the previously shown gene silencing, both CPP/siRNA nanoparticles were capable of inducing RRM2 protein decrease in peritoneal and ovarian endometriotic cells (Figure 2E).

In order to test the therapeutic potential of RRM2 downregulation in different types of endometriosis, cell cycle analysis of transfected endometriotic primary cells was carried out. It became evident that CPP/siRRM2 NPs induced cell cycle arrest in G1/S phase similar to that seen in tumor cells, whereas CPP/siMock did not have any effect on the normal cell cycle when compared to pure siRRM2-treated cells (Figure 2F).

### 3.3. Tumor and Endometriotic Cell Invasiveness Is Reduced after RRM2 and VEGF Knockdown

**Figure 3:**
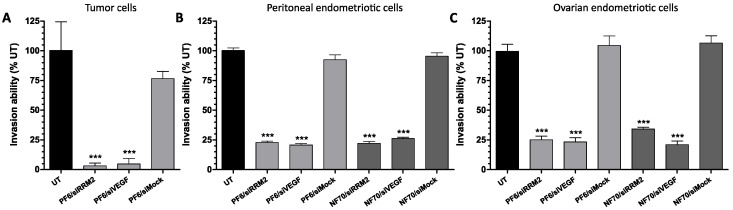
Therapeutic CPP/siRNA NPs reduced cell invasiveness. Invasive capability of (**A**) U-87MG, endometriotic (**B**) peritoneal, and (**C**) ovarian cells through the extracellular matrix. Cells were transferred into Matrigel invasion chambers 48 h after transfection, followed by a 24 h attachment period and a 24 h chemoattraction to FBS. Invaded cells were counted under inverted microscope and results were normalized to UT cells. Data were collected from 3 individual experiments. Error bars represent SEM. *** *p* < 0.001, one-way ANOVA, Tukey post hoc. UT, untreated; FBS, fetal bovine serum.

It is well known that invasion plays an important role in the progression of both cancer and endometriosis [11]. Furthermore, adding another component, an extracellular matrix, into the experiment better mirrors the natural physiological environment of the pathological tissue. Therefore, we proceeded with evaluating the diseased cells’ invasive capability through the Matrigel extracellular matrix after CPP/siRNA treatment and chemoattraction to FBS. The effect of NP transfection to cell phenotype was first investigated in U-87MG tumor cells. As a result, a marked decrease of up to 97% in cell invasiveness was observed after PF6/siRRM2 and PF6/siVEGF treatment (Figure 3A). To continue, in the case of primary peritoneal (Figure 3B) and ovarian (Figure 3C) endometriotic cells, the invasion through the extracellular matrix was also significantly hindered by up to 80%.

### 3.4. PF6 and NF70 Mediate 3D Tissue Penetration of siRNA and Induce Target Gene Knockdown in Tumor and Endometriotic 3D Spheroids

Three-dimensional cell cultures resemble the actual pathological tissue more closely than the monolayer cultures. They have also been shown to be more resistant against therapeutics [29]; therefore, 3D spheroids were further used as a more tissue-like approach.

**Figure 4:**
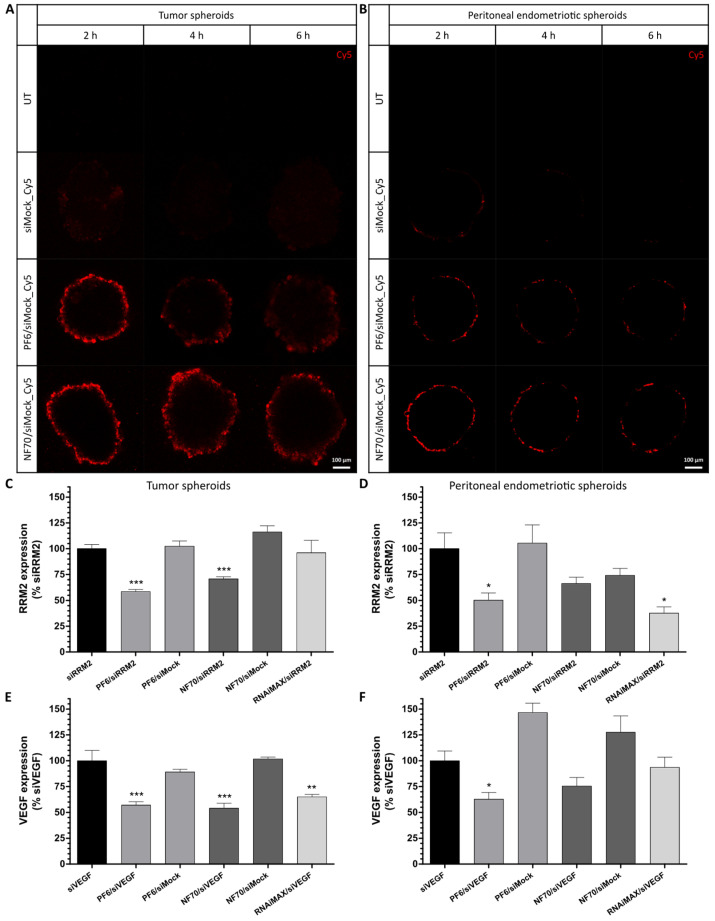
CPP/siRNA NPs exhibited efficient tissue penetration properties and induced therapeutic gene knockdown in 3D spheroid models. (**A**,**B**) Representative confocal images of CPP and fluorescently labelled siMock (red) nanoparticle accumulation into (**A**) U-87MG tumor and (**B**) peritoneal endometriotic spheroids, *n* = 3 independent experiments. Scale bar: 100 µm. (**C**,**D**) *RRM2* and (**E**,**F**) *VEGF* gene expression levels measured in (**C**,**E**) U-87MG and (**D**,**F**) peritoneal endometriotic spheroids using qRT-PCR 48 h after transfection. Data were analyzed by 2^−ΔΔCT^ method using *GAPDH* as an internal control and normalized to relevant pure siRNA groups. Error bars represent SEM of *n* = 7–9 individual samples from independent experiments. * *p* < 0.05, ** *p* < 0.01, *** *p* < 0.001, one-way ANOVA, Tukey post hoc. UT, untreated.

As a first step, tissue penetration of CPP NPs with fluorescently labelled siMock was evaluated in a U-87MG tumor (Figure 4A) and peritoneal endometriotic primary spheroids (Figure 4B). The mid-height z-stack sections of live spheroid confocal imaging are presented. As a result, siRNA accumulation was mainly seen in the cells situated in the outer rim of the spheroids. After confirmation that PF6 and NF70 are capable of transfecting 3D spheroids, we next treated the 3D cultures with therapeutic CPP/siRNA NPs. In case of tumor spheroids, *RRM2* (Figure 4C) and *VEGF* (Figure 4E) were both significantly downregulated by PF6- and NF70-mediated siRNA delivery, whereas lipofection was only significantly effective in knocking down *VEGF* (Figure 4E). When looking at the gene expression levels of peritoneal endometriotic spheroids, therapeutic CPP/siRNA formulations again exhibited strong tendencies towards downregulating the target genes (Figure 4D,F).

### 3.5. Therapeutic CPP/siRNA Nanoparticles Inhibit Tumor Cell Migration

**Figure 5:**
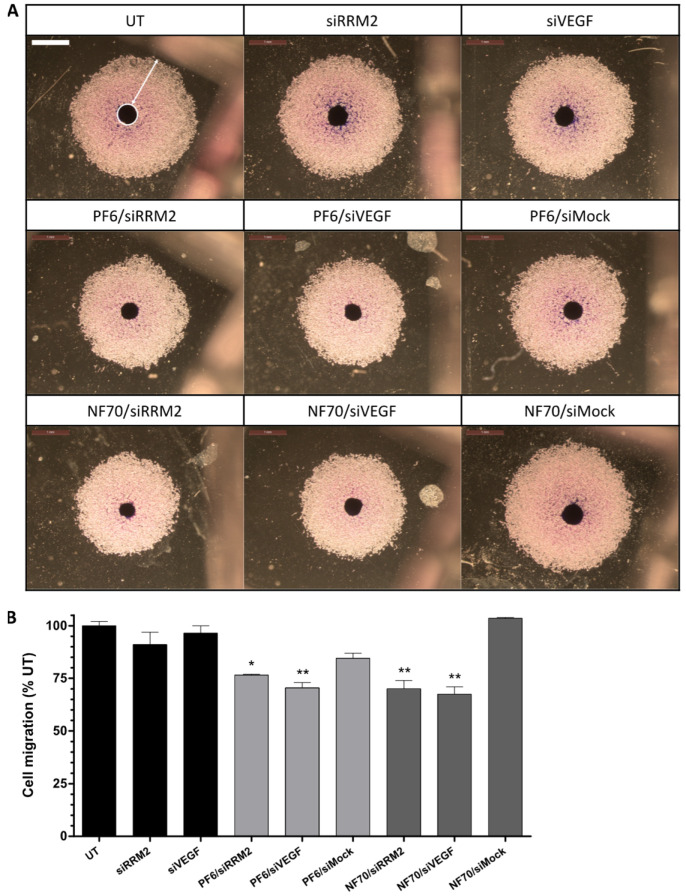
Tumor cell migration from 3D spheroids reduced after therapeutic gene knockdown. (**A**) Representative microscopic images of human fibrosarcoma HT1080 spheroids (white circle) transferred on gelatin-coated plates 48 h after transfection and the surrounding migrating cells (white arrow) 72 h after the transfer. Scale bar: 1 mm. (**B**) Surface area covered by the migrated tumor cells calculated using ImageJ software and normalized to UT group. Error bars represent SEM. * *p* < 0.05, ** *p* < 0.01, one-way ANOVA, Tukey post hoc. Results represent 3 individual experiments. UT, untreated.

The successful knockdown of therapeutic genes in 3D models using CPP/siRNA NPs encouraged us to further investigate the functional effects using disease-specific approaches. One of the crucial steps in the progression of tumors is cancer cell migration. Therefore, we adopted a spheroid-based functional assay [21,22] to investigate the effect of downregulation of *RRM2* or *VEGF* on tumor cell migration. Once transfected spheroids were planted on the gelatin-coated surface, tumor cells started disseminating from the spheroid and migrating over the surface. Spheroids can be seen as a dark dot in the middle of the representative microscopic images (Figure 5A) and the migrated cells as a surrounding light circular area. To illustrate, the spheroid is marked by a white circle and migrated cells by a white arrow on the first UT panel. The surface area covered by the migrated tumor cells measured using ImageJ software showed that the migration of the tumor cells was significantly inhibited in all of the therapeutic CPP/siRNA treatment groups when comparing to untreated spheroids (Figure 5B).

### 3.6. CPP/siRNA NP and Danazol Co-Treatment Synergistically Inhibits Endometriotic Primary Cell Proliferation and Invasion

**Figure 6:**
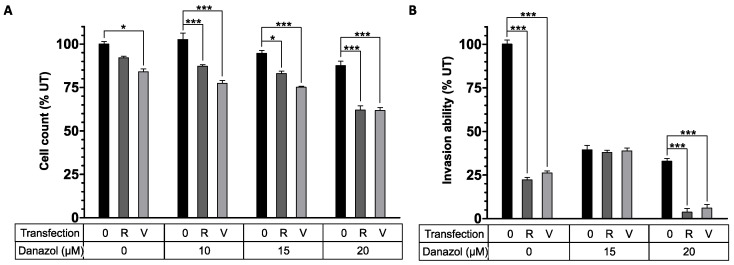
Therapeutic NF70/siRNA NP and danazol co-treatment synergistically reduced endometriotic cell proliferation and invasion. (**A**) Amount of peritoneal endometriotic cells 48 h after co-treatment measured using methylene blue proliferation assay. (**B**) Invasive capability of endometriotic peritoneal cells co-treated with NP–danazol through the extracellular matrix. Cells were transferred into Matrigel invasion chambers 48 h after co-treatment followed by a 24 h attachment period and 24 h chemoattraction to FBS. Invaded cells were counted under inverted microscope. All of the data (**A**,**B**) were collected from 3 independent experiments and results were normalized to UT cells. Error bars represent SEM. * *p* < 0.05, *** *p* < 0.001, two-way ANOVA, Tukey post hoc. Danazol final micromolar concentration is shown. 0—no NP transfection; R—NF70/siRRM2; V—NF70/siVEGF. UT, untreated; FBS, fetal bovine serum.

We postulated that by combining a synthetic steroid, danazol, with an NP treatment strategy, it might be possible to reduce the amount of danazol used and achieve additional synergistic suppression of endometriotic cells. Since PF6 and NF70 had shown similar efficacy in siRNA delivery, only NF70 was used for danazol co-treatment experiments. It has previously been shown, that danazol inhibits the proliferation of endometriotic cells in vitro in a dose-dependent manner [23]. Therefore, to test the NP–danazol co-treatment efficacy, endometriotic cell proliferation rate was measured first (Figure 6A). It was possible to see significant synergistic effect when combining therapeutic NF70/siRNA NP treatment with different danazol concentrations. The effect was best seen at danazol 20 μM concentration, where co-treatment with NPs reduced the cell count approximately 40% when compared to untreated cells, whereas 20 μM danazol alone reduced it only 12% (Figure 6A).

To further study the physiological significance of NP–danazol co-treatment, endometriotic cell invasion capability through the extracellular matrix was measured (Figure 6B). As a result, at danazol 15 μM concentration, the cell invasion was significantly inhibited by up to 60% compared to untreated cells with or without combining danazol with NF70/siRNA NPs. Danazol alone at 20 μM concentration stayed at the same level, but when adding therapeutic NF70/siRNA NPs to danazol during the treatment, a further significant inhibition of invasion was seen; only approximately 5% of the cells were capable of maintaining their invasiveness compared to the untreated condition (Figure 6B).

## 4. Discussion

Gene therapy is a powerful tool that has great potential to treat a wide range of different conditions by transporting genetic material into diseased cells. Thus, biologically active nucleic acids have to cross cell membranes and reach their intracellular targets. To achieve that goal, highly efficient delivery vectors that are harmless to healthy tissue and do not cause severe immune reactions are needed. Cell-penetrating peptides are one class of highly potential non-viral vectors that have proven to be well suited for mediating efficient nucleic acid delivery in vitro and in vivo. CPPs are generally considered safe, easy to synthesize and modify, and they also enable simple non-covalent CPP/siRNA nanoparticle formation [13].

The aim of this work was to test the potential of CPP/siRNA nanoparticles for cancer and endometriosis therapy. For that, in addition to regular monolayer cell cultures, three-dimensional spheroid cultures were used for disease modelling. Cell cultures derived from pathological tissues are the main models used for primary drug testing. However, it has to be taken into consideration that regular monolayer cell cultures do not resemble many of the crucial properties of the pathology, for example cell–cell interactions, tissue structures, intercellular signaling pathways, etc. In order to create more similar conditions to the in vivo environment and to reduce the number of experimental animals used, different in vitro 3D cell cultures have been developed. It has been shown, that in 3D models the gene expression profiles, microenvironment and cell morphology are more similar to the actual situation in the diseased tissue and, therefore, they could be used as an intermediate step between regular two-dimensional monolayer cell culture and in vivo experiments [28].

For conducting gene therapy, two previously described cell-penetrating peptides, PF6 [14] and NF70 [16], were chosen as vectors for siRNA delivery, with *RRM2* and *VEGF* being the target genes. To date, there have been only nine different siRNA-based therapeutic candidates that have reached the clinical evaluation phase for their anticancer activity [30]. One of these nine siRNAs was targeted against RRM2, called CALAA-01 [31], and the second siRNA, with a therapeutic name ALN-VSP02, was used to silence VEGF together with a kinesin spindle protein [32]. Unfortunately, the CALAA-01 clinical trial was terminated after a few years, mainly due to dose-limiting toxicities, whereas several patients’ diseases were progressing during the trial [33]. The ALN-VSP02 trial, on the other hand, was well-tolerated and the patients had 12–18 months tumor stabilization or even complete response for metastatic cancer. Nevertheless, due to the small number of patients and heterogenicity of the tumors, no conclusive therapeutic claims were made and there has been no update on ALN-VSP02 since 2012 [32]. At the same time, the safety and efficacy of other types of RRM2 and VEGF biological or small molecule inhibitors have been extensively studied in clinics, either alone or in combination with other anti-cancer agents, presenting adequate response rates [9,10,34].

Relying on the similarities described for cancer and endometriosis [11,12], we postulated that RRM2 could be suitable for developing endometriosis therapeutics as well. Until now, there have only been a few reports investigating ribonucleotide reductase’s role in endometriosis. One of them is a recent large-scale bioinformatic analysis of previously available microarray datasets, where authors reported *RRM2* being downregulated in endometriosis lesions compared to healthy endometrial tissue in the uterus [35]. Meanwhile they stated that their analysis has limitations by including tissue sample data that were collected from random or unknown menstrual cycle phases. In parallel, it has to be taken into consideration that during the normal proliferative phase of human menstrual cycle the endometrial layer of the uterus indeed has a high proliferation rate of the cells. This is a normal response to steroid hormones produced by the ovaries in order to rapidly prepare for the prospective pregnancy [36]. To add, it has been described that endometriosis lesions also exhibit estradiol-dependent proliferation [37] and, at the same time, ribonucleotide reductase is a crucial enzyme for DNA synthesis and cell proliferation [26]. Furthermore, another report showed the absence of an active form of ribonucleotide reductase in endometriosis lesions only after successful inhibition of their growth in a rat model of endometriosis [38]. Bearing this in mind and in light of the results of this study, we believe RRM2 is vital for endometriosis lesion growth and, therefore, has high potential as a gene target for endometriosis therapy.

The role of VEGF in endometriosis development and progression, on the other hand, has been well described [8]. It was recently shown that the injection of siVEGF and L1 peptide polyplexes directly into endometriosis-like subcutaneous lesions reduced VEGF expression levels and the final lesion volumes in rats [39]. In the meantime, this is one of the few publications that has used peptide-based carriers, together with another report from 2014, where the authors induced apoptosis in spontaneous endometriosis lesions of baboons by combining a pro-apoptotic peptide with an endometriosis-targeting peptide [40]. Unfortunately, there have been no further updates since regarding the clinical relevance of these findings.

According to our knowledge, the current study is the first one that has aimed to develop a synthetic CPP delivery strategy for endometriosis therapy. PF6 and NF70 have both been previously shown to efficiently induce RNA interference after oligonucleotide delivery in in vitro and in vivo conditions [14,16]. PF6 has been extensively studied and its efficacy has also been shown in primary and 3D organotypic cultures [41]. NF70, on the other hand, is a novel peptide, with most recent findings showing it can successfully deliver therapeutic miRNA into primary human keratinocytes and dendritic cells [24]. We investigated the performance of NF70 further in this work and showed it to be highly efficient in transfecting primary 3D cell cultures. Lipofectamine RNAiMAX, on the other hand, did not perform as invariably in inducing gene knockdown in 3D cultures, although it was used at equivalent siRNA concentrations to CPPs, which were ten times higher than the manufacturer’s recommendations. Since Lipofectamine did not have any advantages over CPPs in gene expression studies, rather the opposite, and at the same time the consumption was unreasonably high, it was not used for further functional studies.

Recently, a similar outer rim uptake pattern to the one we saw in 3D tumor and endometriosis spheroids for Cy5-labelled siRNA was shown in ovarian cancer spheroids by delivering mCherry-expressing mRNA by using another CPP, PepFect14 (PF14) [42]. In the same report the authors further achieved in vivo tumor accumulation of analogous PF14/Cy5-labelled mRNA nanoparticles. These findings might suggest that PF6 and NF70 could also be successful in in vivo models of tumor and endometriosis, where the main advantage of using them as cargo delivery vectors would fully be revealed. That is due to both CPPs being pH-sensitive, which may promote nanoparticle uptake in tissues with lower pH, whereas cancer and endometriosis are both considered to have acidic microenvironments [43,44].

Many in vivo preclinical cancer research studies over the years have achieved the induction of therapeutic effects relying solely on the passive accumulation of tested therapeutics in tumors through enhanced permeability and retention (EPR) effects, which are explained by the nature of leaky blood vessels in solid tumors [45]. One recent report has also demonstrated the successful penetration of nontargeted nanoparticles into endometriosis-like lesions in mice [46], which suggests the EPR effect might be present in endometriosis lesions as well. The potential drawback with this type of nontargeted approach is that the payload delivery is not specific to diseased tissue and, therefore, there is a possibility that the drug candidates also affect healthy tissues. It might be the case specifically when the therapeutic molecules may influence the normal physiology of the organism as well. This is supported by the fact that most untargeted drug candidates undergoing clinical trials for cancer therapy are not proving to be highly beneficial when compared to conventional chemotherapy, in addition to the fact that solid tumor permeability is very variable between patients and even in individual patients with multiple tumors [45].

RRM2 is expressed in all cells in the body undergoing mitosis [10], VEGF in all tissues forming blood vessels [9], and the side effects of systemic administration of danazol have been well described [5]. It is remarkable that we showed that it is possible to reduce the amount of danazol administered by combining it with therapeutic NF70/siRNA NPs. Nevertheless, in order to avoid the systemic side effects of therapeutic siRNA or danazol, we believe that further development of the nanoparticles is necessary before proceeding with in vivo validation studies. One option for achieving tissue-specific delivery could be the functionalization of described formulations by targeting moieties like homing peptides previously described for glioblastoma (gHoPe2) [47] or endometriosis (z13) [48]. To further enhance the therapeutic effects, it might be beneficial, for example, to combine siRRM2 and siVEGF during a single treatment to affect several therapeutic targets at the same time or in order to minimize systemic side effects of covalently conjugating danazol to CPPs.

## 5. Conclusions

In summary, we demonstrated that PF6 and NF70 are highly successful in transfecting primary and 3D cell cultures together with the induction of relevant siRNA-mediated therapeutic effects, such as inhibition of migration and invasion in vitro. We also showed that co-treatment with danazol and therapeutic NF70/siRNA nanoparticles synergistically inhibits endometriotic cell proliferation and invasion. All in all, we suggest a novel target, ribonucleotide reductase, for endometriosis therapy, and as a proof-of-concept, we propose a CPP and siRNA-mediated therapeutic approach for cancer and endometriosis, which we strongly believe should be further improved for translational purposes.

## Data Availability

The datasets used and/or analyzed during the current study are available from the corresponding author on reasonable request.

## Supplementary Materials

The following are available online at www.mdpi.com/article/10.3390/pharmaceutics13101618/s1, Figure S1: Peptide purification and mass spectrometry results of (A,C) PF6 and (B,D) NF70. (A,B) Peptides were purified by HPLC on a C4 column using acetonitrile/water gradient containing 0.1% TFA. (C,D) The molecular weight of the peptides was analyzed by MALDI-TOF in positive ion reflector mode using α-cyano-4-hydroxycinnamiic acid as a matrix. (C) PF6 Mw (calculated) = 4418; Mw (found) = 4419.168; (D) NF70 Mw (calculated) = 2929; Mw (found) = 2928.633. Figure S2: Dose titration of siRRM2. *RRM2* gene expression levels in tumor cells measured with qRT-PCR. Data were analyzed by 2^−ΔΔCT^ method using *GAPDH* as an internal control and normalized to untreated cells. Error bars represent SEM, *** *p* < 0.001, one-way ANOVA, Tukey post hoc. UT, untreated. Table S1: siRNA sequences. Table S2: qRT-PCR primer sequences.
