# Supplementary material for: Cell-Penetrating Peptide and siRNA-Mediated Therapeutic Effects on Endometriosis and Cancer In Vitro Models
Source: Pharmaceutics. 2021 Oct 5;13(10):1618. doi: 10.3390/pharmaceutics13101618 (PMC8541686; doi:10.3390/pharmaceutics13101618)
Supplement: Supplementary file 1 [file pharmaceutics-13-01618-s001.zip › pharmaceutics-1340886-supplementary.pdf]

pharmaceutics

Supplementary Materials: Cell-Penetrating Peptide and siRNA-Mediated Therapeutic Effects on Endometriosis and Cancer In Vitro Models

Kristina Kiisholts, Kaido Kurrikoff, Piret Arukuusk, Ly Porosk, Maire Peters, Andres Salumets and Ülo Langel

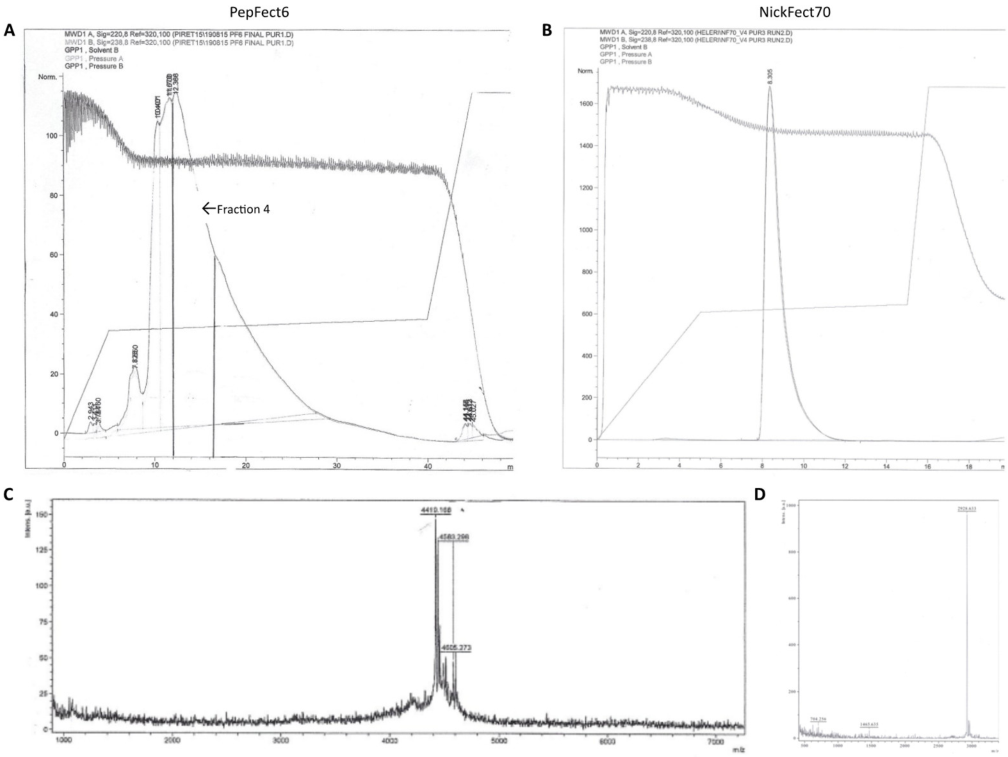

Figure S1. Peptide purification and mass spectrometry results of (**A,C**) PF6 and (**B,D**) NF70. (**A,B**) Peptides were purified by HPLC on a C4 column using acetonitrile/water gradient containing 0.1% TFA. (**C,D**) The molecular weight of the peptides was analyzed by MALDI-TOF in positive ion reflector mode using α-cyano-4-hydroxycinnamiic acid as a matrix. (**C**) PF6 Mw (calculated) = 4418; Mw (found) = 4419.168; (**D**) NF70 Mw (calculated) = 2929; Mw (found) = 2928.633.

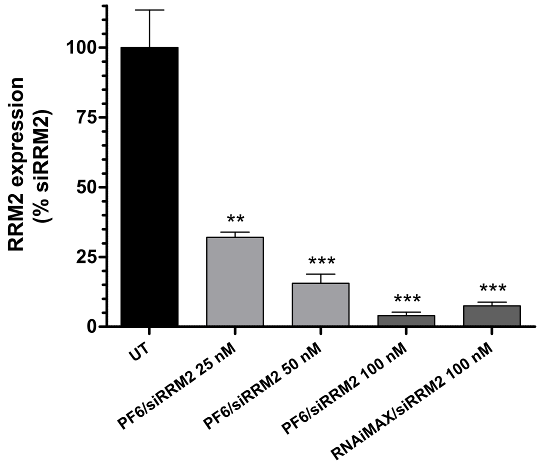

Figure S2. Dose titration of siRRM2. *RRM2* gene expression levels in tumor cells measured with qRT-PCR. Data were analyzed by $2^{-\Delta\Delta CT}$ method using *GAPDH* as an internal control and normalized to untreated cells. Error bars represent SEM, ** $p < 0.01$, *** $p < 0.001$, one-way ANOVA, Tukey post hoc. UT, untreated.

Table S1. siRNA sequences.

siRRM2	Sense	5'-GAU UUA GCC AAG AAG UUC AGA UUa c-3' [a]
	Antisense	5'-GUA AUC UGA ACU UCU UGG CUA AAU CUU-3'
siVEGF	Sense	5'-AUG UGA AUG CAG ACC AAA GAA tt-3' [a]
	Antisense	5'- UUC UUU GGU CUG CAU UCA CAU tt-3' [a]
siLuc	Sense	5'-ACG CCA AAA CAU AAA GAA AG-3'
	Antisense	5'-UUC UUU AUG UUU UUG GCG UCU-3'
siLuc2_Cy5	Sense	5'-Cy5-GGA CGA GGA CGA GCA CUU Ctt-3' [a]
	Antisense	5'-GAA GUG CUC GUC CUC GUC Ctt-3' [a]

[a] Lowercase letters refer to deoxynucleotides.

Table S2. qRT-PCR primer sequences.

RRM2	Forward	5'-TGC TCT CCC TCC GTG TCC CG-3'
	Reverse	5'-CCG GCG GCG TGT TCT CCT TG-3'
VEGF	Forward	5'-CCT GGT GGA CAT CTT CCA GGA GTA-3'
	Reverse	5'-CTC ACC GCC TCG GCT TGT CAC A-3'
GAPDH	Forward	5'-ATG ACC ACA GTC CAT GCC ATC A-3'
	Reverse	5'-CGT CAA AGG TGG AGG AGT GG-3'
